# Supplementary material for: An inflammatory-CCRK circuitry drives mTORC1-dependent metabolic and immunosuppressive reprogramming in obesity-associated hepatocellular carcinoma
Source: Nat Commun. 2018 Dec 6;9:5214. doi: 10.1038/s41467-018-07402-8 (PMC6283830; doi:10.1038/s41467-018-07402-8)
Supplement: Supplementary file 2 — Supplementary Information [file 41467_2018_7402_MOESM2_ESM.pdf]

Supplementary Information

An inflammatory-CCRK circuitry drives mTORC1-dependent metabolic and immunosuppressive reprogramming in obesity-associated hepatocellular carcinoma

Sun *et al.*

Supplementary Figures

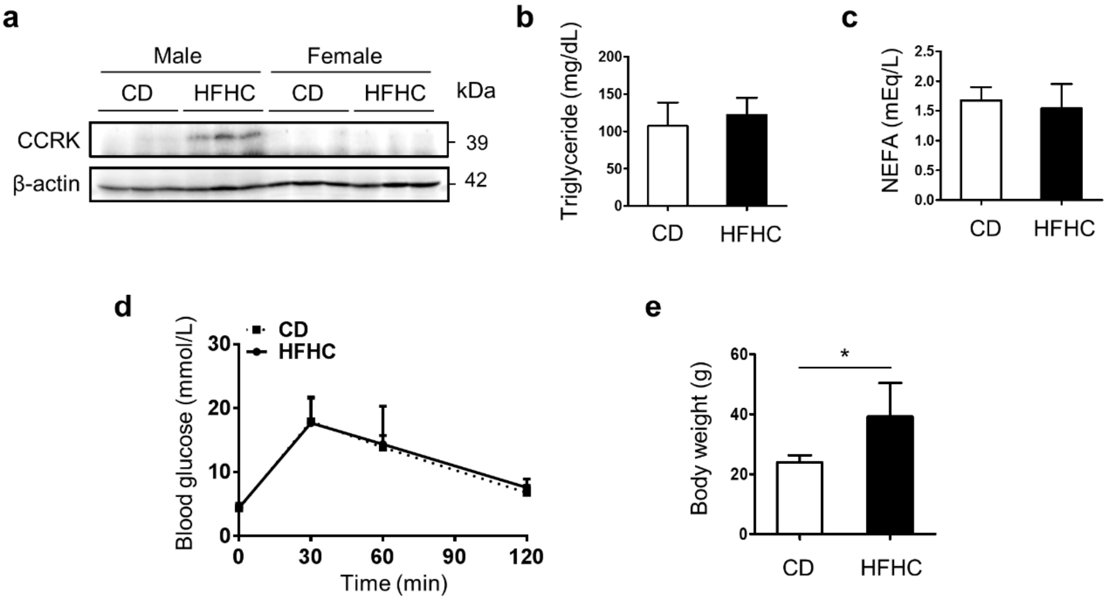

Supplementary Fig. 1 Female mice are not prone to CCRK over-expression or metabolic disorders when fed with HFHC. The HFHC-fed female C57Bl/6 mice did not exhibit (a) CCRK induction, (b-c) triglyceride/NEFA abnormalities, or (d) glucose insensitivity. (e) HFHC increased the body weight of female mice. Data are presented as mean ± SD. *p<0.05 as calculated by unpaired two-tailed Student's *t*-test (e).

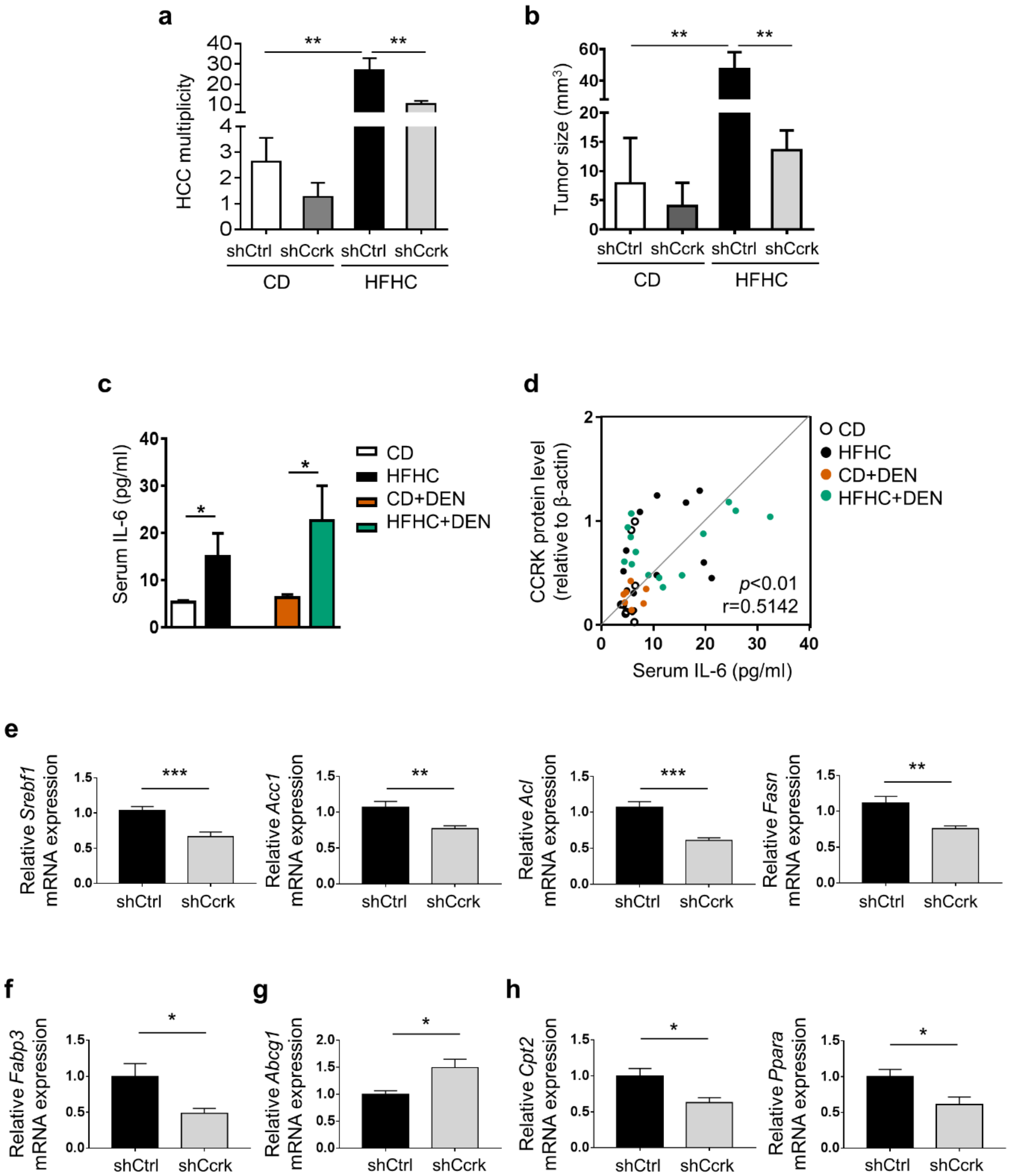

Supplementary Fig. 2 Knockdown of *Ccrk* circumvents obesity-associated metabolic dysregulation and suppresses hepatocarcinogenesis. (a) Tumor multiplicity and (b) tumor sizes were reduced by *Ccrk* knockdown in both HFHC-fed and CD-fed groups, yet no significant difference was observed in the latter case (n≥8). (c) Serum IL-6 levels were increased in the HFHC-fed groups of both NASH and NASH-HCC models. (d) Serum IL-6 levels positively

correlated with CCRK protein expression in both NASH and NASH-HCC models. (e-h) The effects of *Ccrk* knockdown on genes responsible for (e) *de novo* lipogenesis, (f) fatty acid uptake, (g) lipid secretion, and (h) fatty acid beta-oxidation. Data are presented as mean ± SD. *p<0.05; **p<0.01 and ***p<0.001 as calculated by one-way ANOVA followed by Bonferroni post-hoc test (a, b), Pearson correlation (d), and unpaired two-tailed Student's *t*-test (c, e-h).

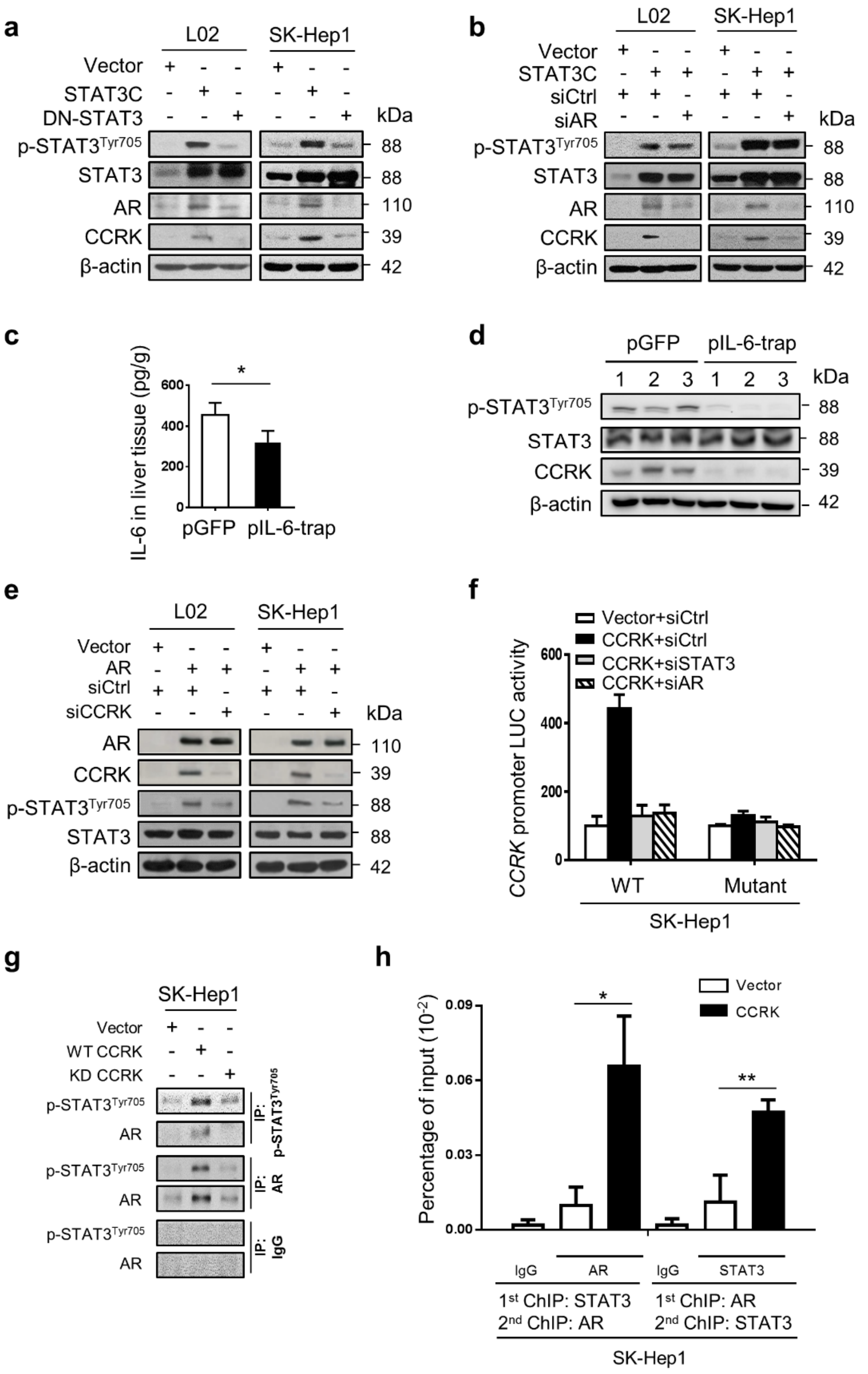

Supplementary Fig. 3 Blockade of IL-6 suppresses STAT3 and AR signaling to down-regulates CCRK expression. (a) Over-expression of constitutively active STAT3 (STAT3C) but not dominant negative STAT3 (DN-STAT3) induced CCRK expression, which (b) was

abrogated by knockdown of *AR* in LO2 and SK-Hep1 cells. (c) Hepatic IL-6 depletion in an orthotopic HCC model using LCP nanoparticles encapsulating plasmids that encode IL-6 protein trap (pIL-6-trap). The nanoparticles with plasmids encoding green fluorescent protein (pGFP) were used as control. ELISA analysis of IL-6 concentration in liver tissue lysates of the pGFP and pIL-6-trap groups. (d) IL-6 neutralization down-regulated STAT3 phosphorylation and CCRK expression. (e) Knockdown of *CCRK* reduced STAT3 phosphorylation in AR-expressing cells. (f) CCRK induced its own promoter activity, which was abolished by deletion of ARE or knockdown of either *STAT3* or *AR* in CCRK-expressing cells. (g) Co-immunoprecipitation of p-STAT3^{Tyr705} and AR in SK-Hep1 cells transfected with WT CCRK, but not in those transfected with empty vector or KD CCRK. IgG is a control for non-specific immunoprecipitation. (h) ChIP-re-ChIP assay demonstrated an increased co-occupancy of STAT3 and AR at the ARE of *CCRK* promotor in SK-Hep1 cells transfected with WT CCRK relative to those transfected with vector only. IgG is a control for non-specific immunoprecipitation. The antibodies used in the 1st and 2nd ChIP are as indicated. Data are presented as mean ± SD. *$p<0.05$ and **$p<0.01$ as calculated by unpaired two-tailed Student's *t*-test (c), and one-way ANOVA followed by Bonferroni post-hoc test (h).

a

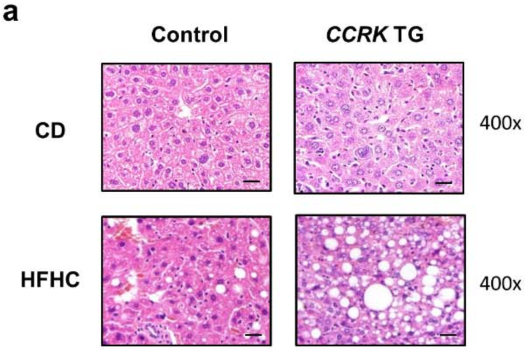

b

Steatosis score	Number of mice	
	Control	*CCRK* TG
0	1 (20%)	0
1	1 (20%)	0
2	2 (40%)	1 (14.3%)
3	1 (20%)	6 (85.7%)

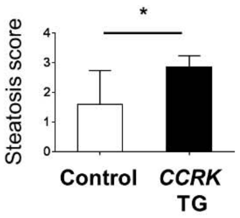

c

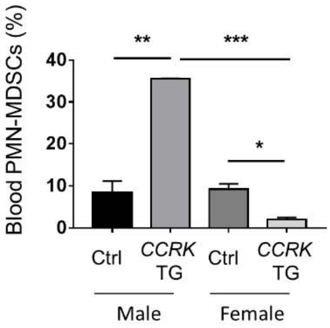

Supplementary Fig. 4 *CCRK* TG mice fed with HFHC diet exhibit enhanced liver steatosis, and PMN-MDSCs are induced in male *CCRK* TG mice. (a-c) Control and *CCRK* TG mice were given tamoxifen treatment and fed with CD or HFHC diet from 3 weeks of age, and then sacrificed at 20 months of age. (a) Representative H&E staining of liver sections of control and *CCRK* TG mice (image magnification=400x, scale bar=20 μm). (b) Liver steatosis score[14] in the TG mice was borderline higher than that in the control group (n≥5). (c) PMN-MDSCs were induced in male but not female *CCRK* TG mice on HFHC diet. Data are presented as mean ± SD. *p<0.05; **p<0.01; ***p<0.001 as calculated by unpaired two-tailed Student's *t*-test (b, c).

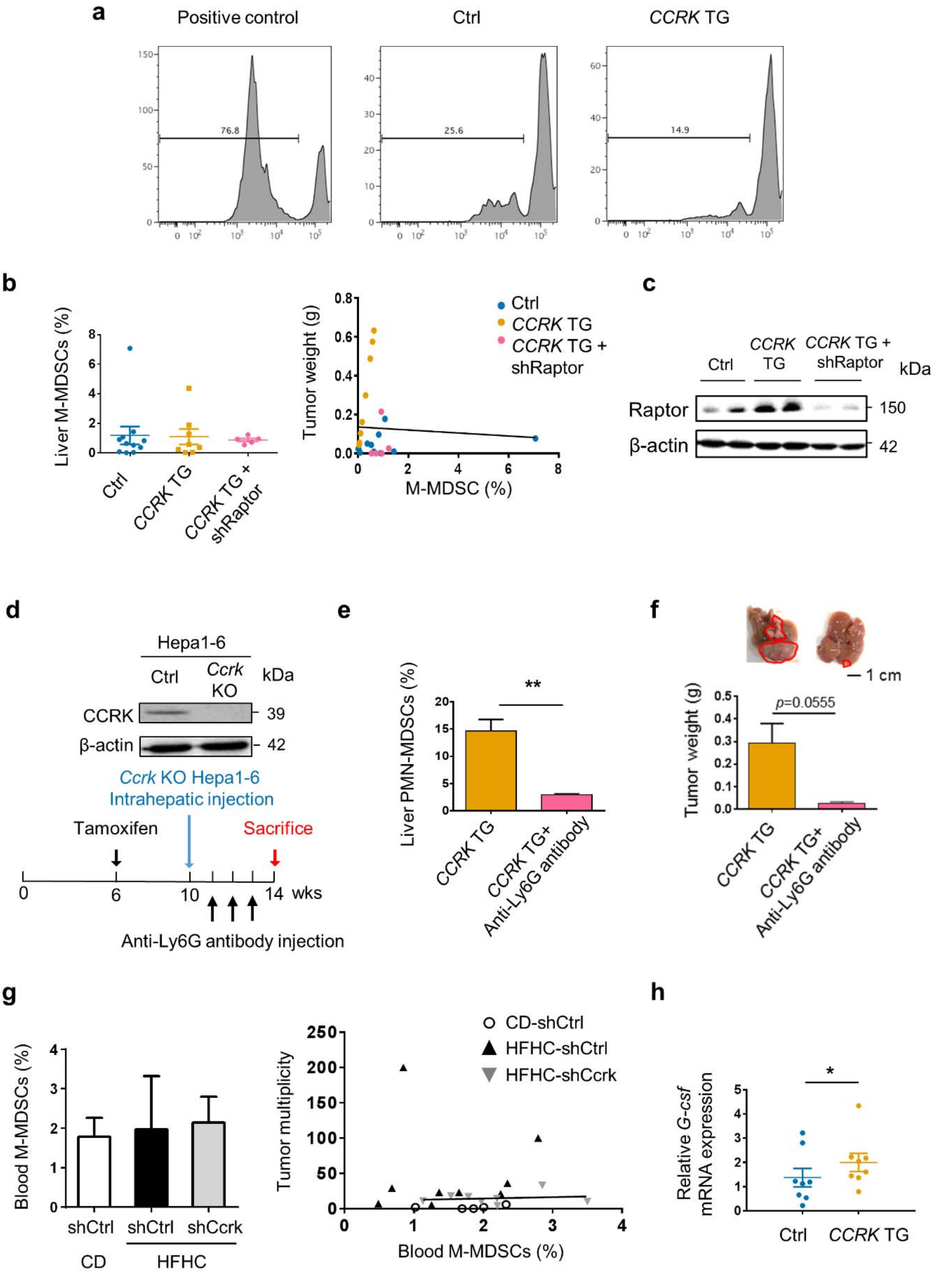

Supplementary Fig. 5 PMN-MDSCs but not M-MDSCs induced by CCRK are responsible for enhanced immunosuppression and tumor growth. (a) PMN-MDSCs from *CCRK* TG mice

exhibited increased T cell suppressive function compared to control mice (n≥3). (b) The percentages of liver-infiltrating M-MDSCs were comparable among control mice, *CCRK* TG mice, and *CCRK* TG mice with *Raptor* knockdown (left). There was no correlation between liver-infiltrating M-MDSCs and tumor weight among the three groups of mice (n≥5). (c) Hepatic Raptor expression was dramatically reduced after shRaptor treatment. (d) Schematic diagram of *CCRK* TG mice subjected to intrahepatic injection of syngeneic *Ccrk* KO Hepa1-6 HCC cells with or without MDSC depletion by anti-Ly6G antibody. (e) Hepatic PMN-MDSC level and (f) tumor weight of *CCRK* TG mice (scale bar=1 cm) were significantly decreased after MDSC depletion (n≥ 4). (g) Blood M-MDSC levels were unchanged in NASH-HCC model, wherein there was no correlation between blood M-MDSCs and tumor multiplicity. (h) *G-csf* was induced in the peri-tumoral liver tissues of *CCRK* TG compared to control mice. Data are presented as mean ± SD. *$p<0.05$ and **$p<0.01$ as calculated by unpaired two-tailed Student's *t*-test (e, h).

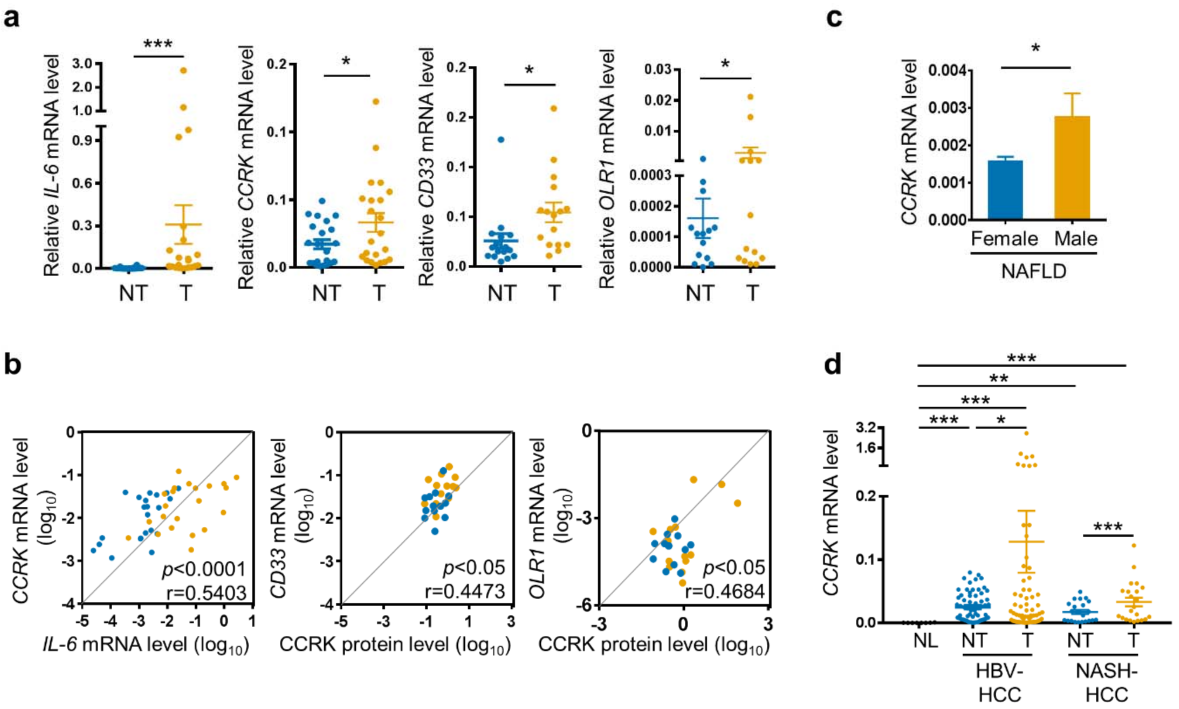

Supplementary Fig. 6 The induction of *IL-6*, *CCRK* and the MDSC markers *CD33* and *OLR1* in tumors compared to matched non-tumor tissues was confirmed by (a) qRT-PCR, which were (b) positively inter-correlated in NASH-HCC patients. (c) *CCRK* mRNA level was elevated in male NAFLD patients (n=13) compared to female NAFLD patients (n=10). (d) *CCRK* mRNA levels were compared in 8 normal liver (NL), 65 and 23 pairs of HBV- and NASH-related HCC tumor (T)/non-tumor (NT) tissues, respectively. Data are presented as mean ± SD. *$p<0.05$; **$p<0.01$ and ***$p<0.001$ as calculated by unpaired two-tailed Student's *t*-test (a, c), Pearson correlation (b), and one-way ANOVA followed by Bonferroni post-hoc test (d).

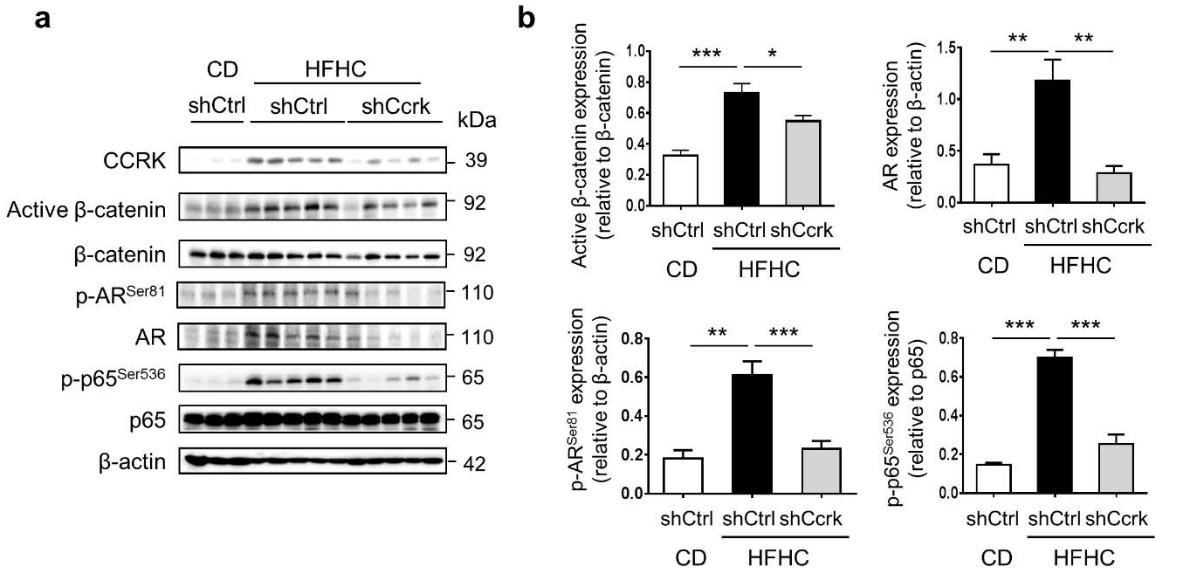

Supplementary Fig. 7 Hepatic β-catenin, AR and NF-κB signaling is regulated by CCRK in NASH-HCC mouse model. (a) The protein expressions of active and total β-catenin, AR, p-AR^{Ser81}, p65 and p-p65^{Ser536} in liver tissues of mice fed with chow diet (CD) and high-fat high-carbohydrate (HFHC) diet with shCtrl or shCcrk lentivirus infection were determined by Western blot (n≥3 per group). (b) Quantification of protein levels is shown in bar charts. β-catenin, β-actin and p65 were used as loading controls. Data are presented as mean ± SD. **p*<0.05; ***p*<0.01 and ****p*<0.001 as calculated by one-way ANOVA followed by Bonferroni post-hoc test (b).

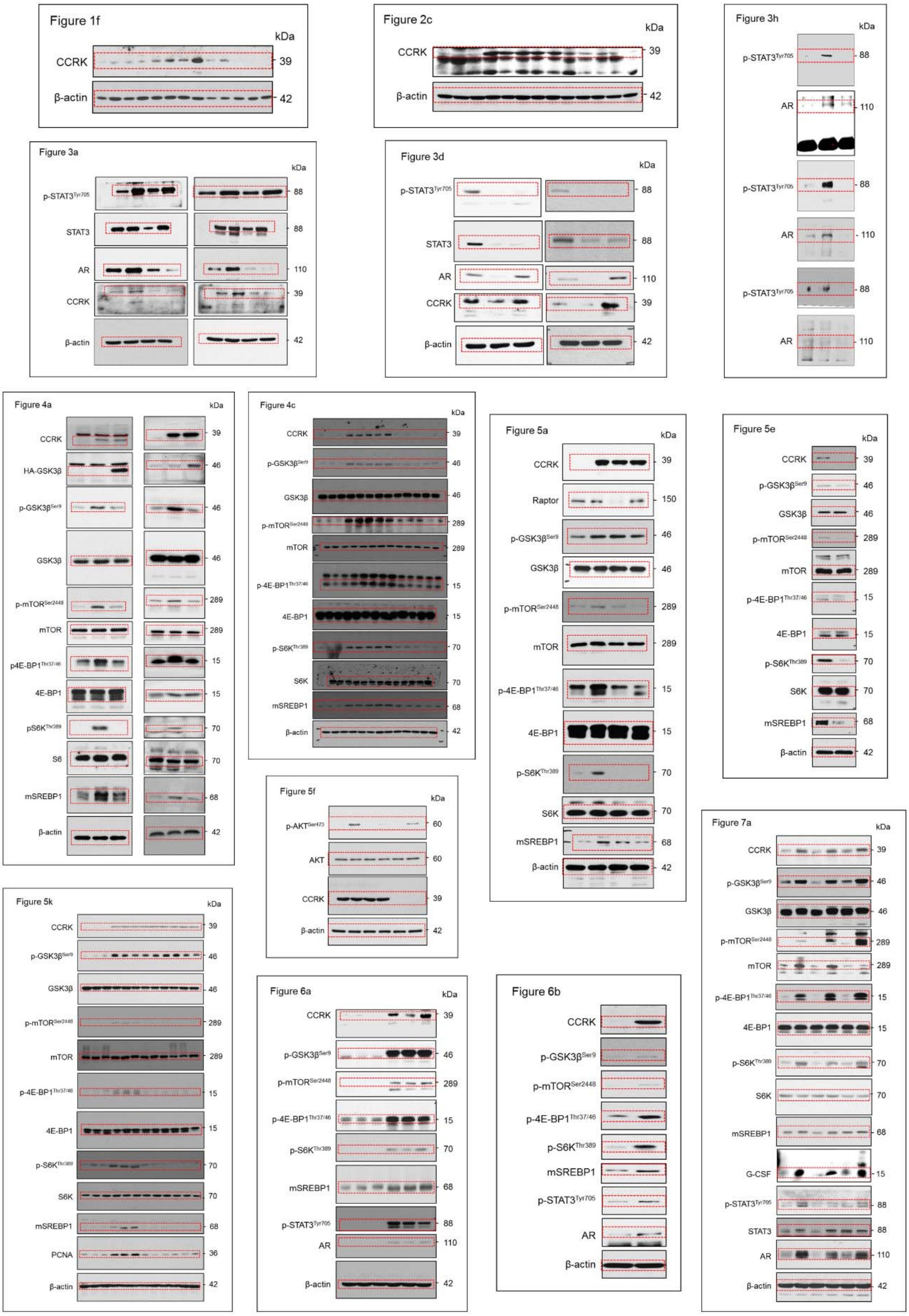

Supplementary Fig. 8 Uncropped and unprocessed Western Blots. Dot line boxes indicate the cropped areas shown in the corresponding figures.

Supplementary Table 1. Clinicopathological information of the NASH-associated HCC patients.

Patient no.	Sex	Age	Hep B	Hep C	NAS*	Steatosis	Lobular inflammation	Ballooning	Diabetes	Hypertension	Dyslipidemia
350	Male	72	negative	negative	1	1	0	0	Yes	Yes	Yes
394	Female	36	negative	negative	0	0	0	0	No	Yes	No
419	Female	78	negative	negative	3	1	1	1	No	Yes	No
465	Male	58	negative	negative	1	0	1	0	Yes	Yes	No
600	Male	64	negative	negative	0	0	0	0	Yes	Yes	No
606	Male	78	negative	negative	1	1	0	0	No	No	No
651	Male	69	negative	negative	1	1	0	0	Yes	Yes	No
666	Female	59	negative	negative	0	0	0	0	No	Yes	Yes
678	Male	58	negative	negative	0	0	0	0	No	Yes	No
696	Male	51	negative	negative	0	0	0	0	Yes	No	No
705	Male	75	negative	negative	1	1	0	0	No	No	No
707	Male	71	negative	negative	0	0	0	0	Yes	Yes	No
717	Male	58	negative	negative	0	0	0	0	Yes	Yes	Yes
741	Female	74	negative	negative	0	0	0	0	Yes	No	No
754	Male	74	negative	negative	1	1	0	0	No	Yes	No
759	Male	60	negative	negative	5	3	1	1	Yes	Yes	No
768	Male	55	negative	negative	0	0	0	0	No	Yes	No
778	Male	59	negative	negative	0	0	0	0	No	Yes	No
784	Male	64	negative	negative	1	1	0	0	Yes	Yes	No
796	Male	67	negative	negative	0	0	0	0	No	Yes	No
797	Male	55	negative	negative	1	0	0	1	Yes	No	No
833	Male	62	negative	negative	0	0	0	0	No	No	No
853	Male	74	negative	negative	1	1	0	0	Yes	Yes	Yes

*NAS: non-alcoholic activity score, which is the sum of steatosis, lobular inflammation and ballooning[1].

Supplementary Table 2. Clinicopathological information of the NAFLD patients.

Patient no.	Sex	Age	Hep B	Hep C	NAS*	Steatosis	Lobular inflammation	Ballooning	Diabetes	Hypertension	Dyslipidemia
200	Female	63	negative	negative	1	1	0	0	Yes	Yes	Yes
346	Female	55	negative	negative	1	1	0	0	No	No	Yes
239	Female	57	negative	negative	2	2	0	0	Yes	No	No
182	Male	57	negative	negative	2	2	0	0	No	No	No
584	Male	41	negative	negative	1	1	0	0	Yes	Yes	No
123	Male	43	negative	negative	1	1	0	0	No	Yes	Yes
169	Male	49	negative	negative	3	3	0	0	No	No	Yes
233	Male	59	negative	negative	1	1	0	0	Yes	Yes	Yes
214	Male	42	negative	negative	1	1	0	0	Yes	Yes	Yes
153	Female	45	negative	negative	5	3	1	1	No	Yes	No
156	Female	61	negative	negative	5	3	1	1	Yes	No	Yes
211	Female	34	negative	negative	5	3	1	1	Yes	No	Yes
216	Female	42	negative	negative	5	2	2	1	Yes	No	Yes
226	Female	59	negative	negative	5	3	1	1	Yes	Yes	No
174	Female	57	negative	negative	5	3	1	1	Yes	No	Yes
198	Female	59	negative	negative	5	3	1	1	Yes	Yes	Yes
183	Male	33	negative	negative	5	3	1	1	No	Yes	Yes
188	Male	38	negative	negative	5	3	1	1	No	No	Yes
142	Male	52	negative	negative	5	3	1	1	Yes	No	Yes
196	Male	57	negative	negative	5	3	1	1	Yes	No	Yes
199	Male	34	negative	negative	5	3	1	1	Yes	Yes	No
240	Male	64	negative	negative	5	3	1	1	No	Yes	Yes
247	Male	52	negative	negative	5	2	2	1	No	No	Yes

*NAS: non-alcoholic activity score, which is the sum of steatosis, lobular inflammation and ballooning[1].

Supplementary Table 3. Primer sequences

Primer name	Species	Sequence 5'-3'	Application
Ccrk-F	mouse	CTGCAGATGCTGCTCAAAGG	qRT-PCR
Ccrk-R	mouse	GCTGGCCTGAGGCACTGAT	qRT-PCR
IL-6-F	mouse	GTAGCTATGGTACTCCAGAGAC	qRT-PCR
IL-6-R	mouse	ACGATGATGCACTTGCAGAA	qRT-PCR
CCRK-F	human	AGACTGGCGAGATAGTTGCC	qRT-PCR
CCRK-R	human	GTGGGAACACAGCCTTCAGT	qRT-PCR
CCRK-promoter(+779)-F	human	CGCAACGGCCCAAAGTAG	ChIP-PCR
CCRK-promoter(+779)-R	human	CCACCCTCCGGCTAACG	ChIP-PCR
G-CSF-F	human	CCACTACACCATCTTCTGGACC	qRT-PCR
G-CSF-R	human	GGTGGATGTGATACAGACTGGC	qRT-PCR
Srebp1-F	mouse	CGACTACATCCGCTTCTTGCAG	qRT-PCR
Srebp1-R	mouse	CCTCCATAGACACATCTGTGCC	qRT-PCR
Acc1-F	mouse	GTTCTGTTGGACAACGCCTTCAC	qRT-PCR
Acc1-R	mouse	GGAGTCACAGAAGCAGCCCATT	qRT-PCR
Acl-F	mouse	AGGAAGTGCCACCTCCAACAGT	qRT-PCR
Acl-R	mouse	CGCTCATCACAGATGCTGGTCA	qRT-PCR
Fasn-F	mouse	CACAGTGCTCAAAGGACATGCC	qRT-PCR
Fasn-R	mouse	CACCAGGTGTAGTGCCTTCCTC	qRT-PCR
Fabp3-F	mouse	AGAGTTCGACGAGGTGACAGCA	qRT-PCR
Fabp3-R	mouse	TTGTCTCCTGCCCGTTCCACTT	qRT-PCR
Abcg1-F	mouse	GACACCGATGTGAACCCGTTTC	qRT-PCR
Abcg1-R	mouse	GCATGATGCTGAGGAAGGTCCT	qRT-PCR
Cpt2-F	mouse	GATGGCTGAGTGCTCCAAATACC	qRT-PCR
Cpt2-R	mouse	GCTGCCAGATACCGTAGAGCAA	qRT-PCR
Ppara-F	mouse	ACCACTACGGAGTTCACGCATG	qRT-PCR
Ppara-R	mouse	GAATCTTGCAGCTCCGATCACAC	qRT-PCR

Reference

1. Brunt EM, Kleiner DE, Wilson LA, Belt P, Neuschwander-Tetri BA, Network NCR. Nonalcoholic fatty liver disease (NAFLD) activity score and the histopathologic diagnosis in NAFLD: distinct clinicopathologic meanings. *Hepatology* **53**, 810-820 (2011).
